# LncRNA-BLACAT1 Facilitates Proliferation, Migration and Aerobic Glycolysis of Pancreatic Cancer Cells by Repressing CDKN1C via EZH2-Induced H3K27me3

**DOI:** 10.3389/fonc.2020.539805

**Published:** 2020-09-23

**Authors:** Xin Zhou, Wei Gao, Huanhuan Hua, Zhimin Ji

**Affiliations:** ^1^Department of Oncology, Linyi People’s Hospital, Linyi, China; ^2^Department of Clinical Laboratory, Linyi People’s Hospital, Linyi, China; ^3^Department of Obstetrics and Gynecology, Kuitun Hospital of Yili Kazak Autonomous Prefecture, Yili Kazak Autonomous Prefecture, Xinjiang, China

**Keywords:** pancreatic cancer, BLACAT1, EZH2, CDKN1C, H3K27, CCNE, glycolysis, oxidative phosphorylation

## Abstract

**Objective:**

To investigate the role of lncRNA-BLACAT1 in promoting H3K27 trimethylation of CDKN1C gene by recruiting EZH2 to regulate CCNE on glycolysis and mitochondrial oxidative phosphorylation of pancreatic cancer (PC) cells.

**Methods:**

Following bioinformatic prediction, EZH2 and BLACAT1 in PC cells were interfered, and cells proliferation, migration and invasion in each group were detected. Western blotting detected the expression of key proteins of mitochondrial complex. The sub-cellular localization of BLACAT1 was tested, followed by testing the binding of CDKN1C and BLACAT1 with EZH2, followed by *in vivo* verification.

**Results:**

Based on bioinformatic prediction, EZH2 and BLACAT1 were highly expressed in PC, while CDKN1C was lowly expressed (all *P* < 0.05). Interference with EZH2 and BLACAT1 inhibited cell proliferation, migration and aerobic glycolysis, and promoted mitochondrial oxidative phosphorylation (all *P* < 0.05). BLACAT1 promoted H3K27 trimethylation of CDKN1C through recruiting EZH2 (all *P* < 0.05). *In vivo* results showed that BLACAT1 interference inhibited tumor formation (all *P* < 0.05).

**Conclusion:**

Interference with BLACAT1 inhibits H3K27 trimethylation of CDKN1C gene by blocking EZH2 recruitment to promote CDKN1C expression and inhibit CCNE expression, thus suppressing PC cell proliferation, migration and aerobic glycolysis, and promoting mitochondrial oxidative phosphorylation.

## Introduction

Pancreatic cancer (PC) is one of the most malignant tumors in the digestive tract (1), which is difficult to diagnose due to its insidious onset and rapid progress (2). Over 80% of the patients with PC are in the advanced stage when they are diagnosed, resulting in the loss of the opportunity of surgical resection and thus a poor prognosis (3, 4). In recent decades, epidemiological investigation supports that the incidence of PC is on the rise at home and abroad (5). At present, the treatment of PC is mainly surgery combined with radiotherapy and chemotherapy (6, 7), yet with unsatisfied clinical effect. It is of great significance to further improve the therapeutic effect of PC by exploring new therapeutic methods.

Long-chain non-coding RNA (lncRNA) has been considered as “noise” in transcription during evolution (8). However, with the development of molecular biology, the role of lncRNA has been paid increasingly more attention. At present, it has been reported that there are many kinds of lncRNA expression differences in human PC tissue/cell, which shows that lncRNA may have potential regulatory role in PC (9). LncRNA-BLACAT1, also known as linc-UBC1, was a novel long non-coding RNA identified in bladder cancer (10). There are several articles referring the role of lncRNA-BLACAT1 such as poor prognostic biomarker for different cancers (11–17), chemoresistance in gastric cancer and NSCLC (18, 19). However, few studies concerning the role of lncRNA-BLACAT1 in the development of PC.

EZH2 is the catalytic active component of PRC2, and its SET domain methylates the 27 lysine H3K27 of H3 histone (20). Besides, H3K27 methylation is related to X-chromosome inactivation, epidermal cell differentiation, and neural stem cell differentiation in mammals (21). Moreover, EZH2 directly interacts with DNA methylation (DNMT1, DNMT3A, and DNMT3B) to maintain the methylation level of DNA, thus inhibiting the expression of some genes (21). Meanwhile, EZH2 alone has no enzyme activity. It must combine two non-catalytic subunits, EEDESC and SUZ2, to obtain strong histone methyltransferase activity (22). A large number of reports have confirmed that EZH2 is regulated at multiple levels, such as mRNA and protein (23). Many studies have found that EZH2 is highly expressed in tumor tissue compared with normal tissue, and its expression level is positively related to the gender and bad prognosis of tumor, which can be acted as an important index for predicting the prognosis of human tumors (24, 25). In PC, EZH2 was found to accelerate the progression of tumorigenesis (26), which might be regulated by E-cadherin (27). Considering that EZH2 is highly expressed in multiple malignant tumors and its expression is positively correlated with metastasis (27), EZH2 may be a new target of tumor therapy. However, whether EZH2 can be used as a molecular marker for early diagnosis and prognosis of PC and the mechanism of EZH2 in the occurrence and development of PC are not clear. Therefore, more extensive and in-depth research and discussion as well as more accurate clinical trial practice are needed in clinical practice.

Most normal tissues get energy through aerobic oxidation of sugar, and glycolysis occurs only in the absence of oxygen (28). The main approach for tumor cells to get energy is glycolysis, and the product of glycolysis is lactic acid (29). This phenomenon is called “Warburg effect,” in other words, malignant tumor cells show high-efficient glucose uptake and produce a large amount of lactic acid under aerobic conditions (30). In recent decades, increasingly more researches support that mitochondrial defects or inactivation may lead to cell adaptation to tumor micro-aerobic conditions, activation of oncogene signals and key enzymes of abnormal metabolism (31). The proposed property can promote the proliferation and invasion of tumor cells and participate in drug resistance. In this regard, tumor cells rely on glycolysis to produce adenosine triphosphate (ATP), which provides us with a new strategy to treat tumor by inhibiting glycolysis and interference with energy metabolism (32, 33). Therefore, it has a broad prospect to explore whether the inhibition of tumor energy metabolism can kill tumor cells and develop glycolysis inhibitors as new anti-tumor drugs. Studies at home and abroad have shown that inhibition of some key enzymes of glycolysis, such as hexokinase, can significantly inhibit the energy metabolism of tumor cells, and then kill tumors (34, 35).

Fatty acid synthesis is identified in most of cancers because of their rapid growth require lipid support (36, 37). ATP-citrate lyase (ALCY) is the first enzyme in this pathway playing the role as converter of citrate to Acetyl CoA, which is followed by catalyzed by Acetyl-CoA carboxylase (ACC) to malonyl-CoA. Both Acetyl-CoA and malonyl-CoA are assembled with fatty acid synthase (FASN), and thus generated palmitic acid (38). ACC is the core kinase to sense the energy status in physiological condition and was regulated by LKB1/AMPK (39). In PC, the pathway of fatty acids synthesis is highly activated (40). ACLY inhibition is found to reduce the PC growth in mouse xenograft (41). AMPK inhibition cooperated with MAPK blockage has been showed to significantly inhibit the proliferation of Ras-mutant cancer cells (42). Besides, FASN was also found highly expressed in PC patients (43) and correlated with poor survival (44). Taken together, fatty acid metabolism is aberrantly activated in PC, which provide lipid supply for cancer cell proliferation.

G1/S-specific cyclin-E1 (CCNE) is a member of cyclin family, which cooperate with CDK2 to regulate the eukaryotic cell cycle (45). The role of CDKs in regulating energy metabolism especially glycolysis is noticed. Wang reported in Nature, 2017 that cyclin D3-CDK6 is able to regulate glycolytic pathways (46), which is followed by the emerging studies talking about the directly or indirectly regulation of metabolism by cyclin-CDK counterparts. With respect to CCNE, its cooperator, CDK2 was found to be related with glucose tolerance by FOXO1 phosphorylation (47). These reports reminds us the potential linkage between cyclin and metabolism.

On the basis of the above understanding, the present study was thus conducted to investigate the role of lncRNA-BLACAT1 in promoting the H3K27 trimethylation of CDKN1C gene by recruiting methyltransferase EZH2 to regulate the expression of CCNE gene on glycolysis and mitochondrial oxidative phosphorylation of PC cells.

## Materials and Methods

### Ethics Statement

All the patients in this study signed informed consent, and the study was approved by the Ethics Committee of Linyi People’s Hospital and followed the Declaration of Helsinki. The animal experiments were performed in strict accordance with the recommendations in the Guide for the Care and Use of Laboratory Animals of the National Institutes of Health. The protocol of animal experiments was approved by the Institutional Animal Care and Use Committee of Linyi People’s Hospital. The animal experiments were conducted based on minimized animal number and the least pains on experimental animals.

### Bioinformatic Analysis

The expression level of EZH2 and survival status of patients with PC were obtained by using GEPIA^1^ and UALCAN databases^2^. The upstream lncRNA of EZH2 was obtained by searching RAID database^3^. GSE16515 chip was obtained by searching GEO database^4^. Using R language “limma” package, the difference analysis was carried out with normal samples as the control, and the differential p value was corrected by false discovery rate (FDR), with |logFC| > 2 (FC: fold change) and FDR < 0.05 as the criteria for screening differential genes. KEGG database^5^ was then used to search for downstream genes and related pathways of CDKN1C.

### Objects of Study

From January 2018 to January 2019, 42 cases of PC and paracancerous tissues were collected for study, all of which were confirmed by histopathology. Of the enrolled patients, the mean age was 54.52 ± 7.51 years old, including 20 males and 22 females, 11 highly differentiated, 13 moderately differentiated, 18 poorly differentiated, 25 lymph node metastasis, and 17 non-lymph node metastasis. Besides, according to the UICC staging method (48) the clinical stages were 14 cases in stage I + II and 28 cases in stage III. Inclusion criteria: patients without drug therapy, chemotherapy, radiotherapy and immunobiological treatment, etc. Exclusion criteria: patients who had previous treatment history of drug therapy, chemotherapy, radiotherapy and immunobiological treatment, etc. All patients in the experiment had informed consent to the experiment, and signed the informed consent form. The study was approved by the Ethics Committee of Linyi People’s Hospital, which was in line with the Declaration of Helsinki.

### Immunohistochemical Staining

Paraffin embedded sections of tumor tissues in each group were used for immunohistochemical analysis. After dewaxing, dehydration with gradient alcohol, washing with tap water for 2 min, the next steps were soaking for 20 min in 3% methanol H_2_O_2_, 2 min in distilled water, 3 min in 0.1M PBS, followed by water-bath repairing in antigen repair solution, and cooling with tap water. After that, normal goat serum blocking solution (C-0005, Shanghai Haoran Bio Technologies Co., Ltd., Shanghai, China) was dropped on the tissue slices, and then the slices were placed at room temperature for 20 min with the liquid on the slide dried by spinning. Then, primary antibodies of CDKN1C (ab75974, 1: 250) and CCNE (ab33911, 1:500) were added on the tissue slices and incubated overnight at 4°C. The slices were washed three times in 0.1M PBS (5 min/time), followed by the addition of goat anti-rabbit IgG secondary antibody, and the tissue slices were placed at 37°C for 20 min and washed three times with PBS (5 min/time). Subsequently, horseradish peroxidase labeling streptomyces ovalbumin working solution (0343-10000U, Yimo Biotechnology Co., Ltd., Beijing, China) was added for incubation at 37°C for 20 min, followed by washing in 0.1M PBS (three times in total, 5 min/time). Afterward, with development using DAB (ST033, Guangzhou Weijia Technology Co., Ltd., Guangzhou, China) and washing with water after that, re-staining was performed with hematoxylin (PT001, Shanghai Bogoo Biotechnology Co., Ltd., Shanghai, China) for 1 min, and then bluing was performed with 1% aqueous ammonia after washing with water, followed by another washing with water. In the next steps, dehydration was carried out with gradient alcohol at certain concentration, transparency processing with xylene, and sealing with neutral resin. During observation under microscope, 5 high magnification visual fields were selected randomly for each slice with 100 cells in each field of vision (positive cells < 10%, negative, positive cells ≥10% and <50%, positive, positive cells >50%, strong positive) (49).

### qRT-PCR

TRIzol (Invitrogen, Calsbad, CA, United States) was used to extract total RNA from tissues and cells. Nanodrop2000 micro-ultraviolet spectrophotometer (1011U, nanodrop, United States) was used to detect the concentration and purity of total RNA. Reverse transcription was conducted to generate cDNA according to the instruction of TaqMan MicroRNA Assays Reverse Transcription primer (4427975, Applied Biosystems, United States). The primers of EZH2, BLACAT1, CDKN1C, and CCNE were designed and then synthesized by Takara company (Table 1). ABI 7500 quantitative PCR instrument (7500, ABI, United States) was used for real-time fluorescence quantitative PCR detection. The reaction conditions were pre-denaturation at 95°C for 10 min, denaturation at 95°C for 10 s, annealing at 60°C for 20 s, and extension at 72°C for 34 s, with a total of 40 cycles. The relative transcription level of target gene was calculated by relative quantitative method (2-ΔΔCT method) with GAPDH as internal reference: ΔΔCt = ΔCt experimental group-ΔCt control group, ΔCt = Ct (target gene) – Ct (internal reference), relative transcription level of target gene mRNA = 2-ΔΔCt. Each experiment was repeated three times.

**TABLE 1:** Primer sequences of qRT-PCR.

Gene	Primer sequences (5′-3′)
EZH2	F: 5′-GGGAAGAAATCTGTGTGTTGGAA-3′ R: 5′-TGTGTTGGAAAATCCAAGTCA-3′
BLACAT1	F: 5′-GAATCGGACAAGGAGGGAAGA-3′ R: 5′-TGGTGGTGATGAGTTTAGATGCT-3′
CDKN1C	F:5′-CTGCACTATCTCTCCATGTTC-3′ R:5′-GCGATCTCACACTTGTTCA-3′
CCNE	F:5′-GCCGAGCGGTAGCTGGTC-3′ R:5′-GGGCTGCTGCTTAGCTTGTAAA-3′
GAPDH	F:5′-AACGGATTTGGTCGTATTGGG-3′ R:5′-TCGCTCCTGGAAGATGGTGAT-3′

### Western Blot

Total protein in tissues and cells was extracted by RIPA lysate containing PMSF, incubated on ice for 30 min, and centrifuged at 4°C (8,000 *g* for 10 min) to collect the supernatant. BCA kit was used to detect the total protein concentration. An amount of 50 μg protein was dissolved in 2 × SDS sample buffer and boiled at 100°C for 5 min. The above samples were then used for SDS-PAGE gel electrophoresis. After that, the protein was transferred to PVDF membrane by wet transfer method, followed by sealing with 5% skimmed milk powder at room temperature for 1 h. Then, the diluted rabbit anti-EZH2 (ab186006, 1:1000, Abcam, Cambridge, United Kingdom), CDKN1C (ab75974, 1:500, Abcam, Cambridge, United Kingdom), CCNE (ab33911, 1:1000, Abcam, Cambridge, United Kingdom), COMPLEXII-SDHB (ab178423, 1:2000, Abcam, Cambridge, United Kingdom), COMPLEXV-ATP5A (ab14748, 1:1000, Abcam, Cambridge, United Kingdom) and COMPLEXIV-COXII (ab110258, 1:1000, Abcam, Cambridge, United Kingdom) were added in the PVDF membrane and incubated overnight at 4°C, with GAPDH (ab9485, 1:2500, Abcam, Cambridge, United Kingdom) as the internal reference. After washing with TBST three times (10 min each), the membrane was incubated with HRP labeled goat anti-rabbit IgG H&L (HRP) (ab97051, 1:2000, Abcam, Cambridge, United Kingdom) for 1 h. After TBST rinsing, the membrane was placed on a clean glass plate. Then, solution A and solution B of equal amount in the ECL fluorescence test kit (BB-3501, Ameshame, United Kingdom) were taken and mixed in dark, then dripped onto the membrane and placed in the gel imager for imaging. Photography was performed with Bio-Rad image analysis system (Bio-Rad, United States) and analysis was carried out based on Quantity One v4.6.2 software. The relative protein content was expressed by the gray value of corresponding protein bands/the gray value of GAPDH protein bands. The experiment was repeated three times to take the average value.

### Cell Culture, Grouping and Transfection

Human pancreatic duct epithelial cells (HPDE) and PC cell lines BxPC-3, Capan-1, PANC-1, CFPAC-1, and Hs766T were purchased from ATCC. After resuscitation, DMEM medium (Gibco, United States) containing 10% fetal bovine serum (Gibco, United States) was cultured in an incubator with 5% CO_2_ and saturated humidity at 37°C (Thermo Fisher Scientific, United States). When the cell density reached 90%, 0.25% trypsin (T1300, Solarbio, China) was used for trypsinization and then subculture at the ratio of 1:3. The expression of EZH2 in human PC cell line was detected by qRT-PCR, and then it was used for subsequent cell experiments.

The human PC cell line PANC-1 in logarithmic growth phase was transfected, and the experimental groups were as follows: sh-NC group (Transfection of and interference with EZH2 negative plasmid), sh-EZH2 group (Transfection of and interference with EZH2 plasmid), sh-NC group (Transfection of and interference with BLACAT1 negative plasmid), sh-BLACAT1 group (Transfection of and interference with BLACAT1 plasmid), oe-NC group (Transfection of overexpressed BLACAT1 negative plasmid), oe-BLACAT1 group (Transfection of overexpressed BLACAT1 plasmid), sh-BLACAT1 + sh-NC group (Transfection of and interference with BLACAT1 plasmid and CDKN1C negative plasmid), sh-BLACAT1 + sh-CDKN1C group (Transfection of and interference with BLACAT1 plasmid and CDKN1C plasmid), sh-CDKN1C + sh-NC group (Transfection of and interference with CDKN1C plasmid and CCNE negative plasmid), and sh-CDKN1C + sh-CCNE group (Transfection of and interference with CDKN1C plasmid and CCNE plasmid).

All transfection sequences were synthesized by Sangon Biotech (Shanghai) Co., Ltd. Cell transfection: the cells were subcultured 1 day before transfection and inoculated into the 6-well plate with 1 × 10^5^ cells per well. When the cells reached 70–80% fusion, cell transfection was carried out with reference to the instruction of Lipofectamine 2000 transfection reagent (11668019, Invitrogen, Carlsbad, CA, United States). The 100 pmol sequence was diluted with 250 μL serum-free DMEM at final concentration of 50 nM, mixed gently, and incubated at room temperature for 5 min. Then, the 5 μL Lipofectamine 2000 was diluted with 250 μL serum-free DMEM, mixed gently and incubated at room temperature for 5 min. The above mixtures were incubated at room temperature for 20 min and added to the cell culture well. The transfected cells were cultured in 5% CO_2_ incubator at 37°C for 6–8 h, and then transferred to a complete medium, followed by culture for 24∼48 h for subsequent usage.

### EDU Assay

The cells to be tested were inoculated into the 24-well plate. Cells in each group were made in three replicates. EdU was added into the culture medium at the concentration of up to 10 μmol/L, followed by incubation for 2 h. After absorbing the medium, the cells were fixed at room temperature for 15 min with PBS solution containing 4% paraformaldehyde, washed twice with PBS containing 3% BSA, incubated at room temperature for 20 min with PBS containing 0.5% Triton-100, and washed twice with PBS containing 3% BSA. After that, 100 μL Apollo^®^ 567 (Guangzhou RiboBio Co., Ltd.) was added per well, incubated at room temperature in dark for 30 min, then washed twice with PBS containing 3% BSA, followed by the addition of DAPI for nucleus staining for 5 min, and washed three times with PBS. After sealing, the number of positive cells was observed and recorded in each field of vision under fluorescence microscope (model: FM-600, Shanghai Pudan Optical Chemical Instrument Co., Ltd.). Total cells observed were blue in appearance and the positive cells were red under the microscope. Three fields were randomly selected for each well to calculate the proportion of EdU positive cells. EdU positive rate = (EdU number of positive nuclei/total number of positive nuclei) × 100%.

### Transwell Assay

Migration assay: PC cells in the logarithmic growth stage were starved for 24 h, collected and re-suspended to adjust the final cell concentration at 2 × 10^5^/ml. Then, 0.2ml suspension was added to the upper chamber of Transwell, and 700 μl pre-cooled DMEM cell culture solution containing 10% FBS was added to the lower chamber. Cells were cultured in a cell incubator containing 5% CO_2_ at 37°C. After 24 h of incubation, the Transwell chamber was removed, cells on the upper chamber and basement membrane were wiped with a wet cotton swab, fixed with methanol for 30 min, and stained with 0.1% crystal violet for 20 min. After rinsing with running water, turning upside down, cells were dried naturally, and then observed and photographed under inverted microscope. The experiment was repeated three times.

Invasion assay: ECM glue (MERCK) was placed at 4°C overnight, which was then diluted the next day (pre-cool all gun heads and chambers on ice for half an hour before the experiment) with serum-free medium at the ratio of 1:9 to the final concentration of 1mg/ml. An amount of 40 μl ECM glue was added to the polycarbonate film of each 24-well Transwell upper chamber, and incubated in an incubator containing 5% CO_2_ and at 37°C for 5 h, so that the ECM glue could polymerize to form a gel. After absorbing and discarding the superfluous liquid, pure DMEM medium was added 70 μl per chamber and incubated in 37°C incubator for 0.5 h, followed by rehydration of the matrix glue, and removal of the superfluous medium for further usage. Cells in each group were digested, centrifuged and re-suspended in DMEM medium without FBS for 24 h after serum withdrawal and starvation. The final cell concentration was adjusted to 2.5 × 105/ml. Afterward, 0.2 ml suspension was collected and added to the upper chamber where the basement membrane had been hydrated, followed by the addition of 700 μl pre-cooled DMEM medium containing 10% FBS to the lower chamber. It was cultured at 37°C in a 5% CO_2_ saturated humidity incubator for 24 h. The chamber was removed, and the cells were removed from the chamber and basement membrane with a wet cotton swab, fixed with methanol for 30 min, stained with 0.1% crystal violet for 20 min, and dried upside down. Consequently, cells were observed and photographed under inverted microscope. The experiment was repeated three times.

### Determination of Lactate/Glucose by Hexokinase Method

According to the instructions of hexokinase (HK) kit (product No.: BC0740, Solarbio, Beijing, China), PC cells in each group were cultured in equal amount of fresh medium, and the content of glucose and lactic acid in the supernatant was detected. After that, further analysis was focused on glucose uptake and lactate production.

### Comparative Analysis of ATP

Pancreatic cancer cells were collected and re-suspended in PBS at the cell density of 1 × 10^6^ cells/ml. ATP lysis buffer was added to the cells and incubated on ice for 30 min. The cell lysate was centrifuged at 12,000 r/min for 10 min, and then the ATP level in the supernatant was detected by ATP detection kit (Beyotime, Jiangsu, China). Pi-102 fluorescence luminometer (Hygiena, New York, NY, United States) was used for the measurement of Bioluminescence, ATP content in cells was calculated by ATP standard and standardized by BCA protein assay.

### FISH

FISH was used to detect the location of BLACAT1 in PC cells according to the instruction of RiboTM lncRNA FISH Probe Mix (Red) (Guangzhou RiboBio Co., Ltd., Guangzhou, China). According to the specific method of processing, after placing the cover glass in the 6-hole culture plate, PC cells were collected and cultured for 1 day to make the cell fusion rate about 80%. Then, the slide was taken out, washed with PBS and added with 1 mL 4% paraformaldehyde for fixation at room temperature, treated with protease K (2 μg/mL) glycine and glyophthalein, added with 250 μL pre-hybridizing solution, and then incubated at 42°C for 1h. After absorbing the pre-hybridizing liquid, 250 μL of hybridizing liquid containing probe (300 ng/mL) were added and hybridized overnight at 42°C. After PBST cleaning for three times, DAPI (1:800) staining solution diluted with PBST was added to stain the nucleus, followed by the addition to 24-well culture plate, staining for 5 min, and PBST cleaning for three times, 3 min each time. After being sealed with anti-fluorescence quenching agent, five different visual fields were selected under the fluorescence microscope (Olympus, Japan) for observation and photography (50).

### RNA-Binding Protein Immunoprecipitation

The binding of BLACAT1 to EZH2 was detected by RIP Kit (Millipore, United States), and the cells to be tested was washed with pre-cooled PBS. After discarding the supernatant, cells were lysed with RIPA lysate (P0013B, Beyotime) of equal volume in ice bath for 5 min, and centrifuged at 4°C for 10 min (14,000 rpm). Following co-precipitation of cell extract and antibody, 50 μL magnetic beads were collected from each co-precipitation reaction system, washed and then suspended in 100 μL RNA-binding protein immunoprecipitation (RIP) Wash Buffer. According to the experimental grouping, 5 μg antibody was added to incubate for binding. After cleaning, the bead-antibody complex was re-suspended in 900 μL RIP Wash Buffer and incubated overnight at 4°C with 100 μL cell extract. The sample was placed on the magnetic base to collect the bead-protein complex. After digestion with protease K, RNA was extracted for subsequent PCR detection. The antibody applied in RIP was rabbit anti-EZH2 (1:500, Abcam, ab186006, United Kingdom), which was mixed at room temperature for 30 min, and IgG (ab109489, 1:100, Abcam, United Kingdom) was used as negative control.

### RNA Pull Down

*In vitro*, T7 RNA polymerase (Ambion, United States) was used to transcribe into BLACAT1 fragment, and then DNase I (Qiagen, Germany) was used for treatment according to the instruction of using RNeasy Plus Mini Kit (Qiagen, Germany), and RNeasy Mini Kit was used for purification. The purified RNA 3′end was labeled with biotin RNA marker mixture (Ambion, United States). Then, 1 μg labeled RNA was heated to 95°C in RNA structure buffer (10 mmol/L Tris pH7, 0.1 mol/L KCl, 10 mmo/L MgCl_2_) for 2 min, then transferred to ice for 3 min, and then left at room temperature for 30 min to form a proper secondary structure. An amount of 3 μg of PC cells were added to cell lysate (Sigma, United States) at 4°C for 1 h. The lysate was centrifuged at 4°C for 10 min (12,000 × *g*), and the supernatant was collected and transferred to the RNase-free centrifuge tube. Then, 400 ng biotinylated RNA was added to 500 μL RIP buffer and incubated with lysate at room temperature for 1 h, then streptavidin beads were added to each binding reaction and incubated at room temperature for 1 h. Finally, after washing with RIP buffer for five times and addition of 5× sample buffer, the eluted EZH2 protein was incubated at 95°C for 5 min, and then detected by western blot.

### Chromatin Immunoprecipitation

Pancreatic cancer cells of 70–80% fusion were added with 1% formaldehyde, and fixed at room temperature for 10 min to fix and cross-link the DNA and protein in cells. After cross-linking, cells were randomly broken by ultrasonic treatment for 10 s each time, with time interval of 10 s, in a total of 15 cycles, so as to break the cells into fragments of appropriate size. After centrifugation at 4°C (13,000 rpm), the supernatant was collected and divided into three tubes, with the addition of RNA polymerase II (ab109489, 1:100, Abcam, United Kingdom) as the positive control antibody, the IgG (ab109489, 1:100, Abcam, United Kingdom) as the negative control antibody of normal mice and the mouse anti-H3K27me3 (1:100, ab4729, Abcam, United Kingdom) as the target protein specific antibody, followed by overnight incubation at 4°C. The endogenous DNA protein complex was precipitated by Protein Agarose/Sepharose, the supernatant was removed after transient centrifugation, and the non-specific complex was washed, followed by overnight cross-linking at 65°C. Then, the DNA fragments were extracted, purified and recovered by using phenol/chloroform. Finally, the binding of CDKN1C to H3K27me3 was detected by CDKN1C promoter specific primers.

### Tumorigenesis Experiment in Nude Mice

Thirty-two female nude mice (5–6 weeks) were provided by Shanghai Slake Experimental Animal Co., Ltd., which were raised in an experimental environment with constant temperature (25∼27°C) and constant humidity. With the growth rate reaching 80–90%, stable transfected cells were digested and centrifuged, washed by PBS for 2–3 times and then re-suspended for counting. With the adjustment of cell density to 1 × 10^7^ cells/mL, 20 μL cell suspension were taken and inoculated under the armpit of nude mice, with eight mice in each group. Then, the size of transplanted tumor was measured every week to record the volume of transplanted sarcoma and draw the growth curve. The formula was (a × b^2^)/2 (a was the longest diameter of the tumor, and b was the shortest diameter). Nude mice were killed with CO_2_ 5 weeks later. All experimental operations were in accordance with the International Convention on the ethics of experimental animals, in line with the relevant national regulations.

### Statistical Analysis

SPSS 21.0 (IBM SPSS Statistics, Chicago, IL, United States) was used for statistical analysis. All data were in accordance with the analysis of normal distribution and the homogeneity of the variance. The measurement data were expressed by mean ± standard deviation, and paired *t*-test was used for comparison between cancer tissue and adjacent tissue. Independent sample *t*-test was used between groups in the remaining groups, one-way analysis of variance (ANOVA) was used for comparison among multiple groups, and Tukey’s test was used for *post hoc* test. *P* < 0.05 meant that the difference was statistically significant.

## Results

### EZH2 Overexpression in PC Tissues and Cells

**FIGURE 1:**
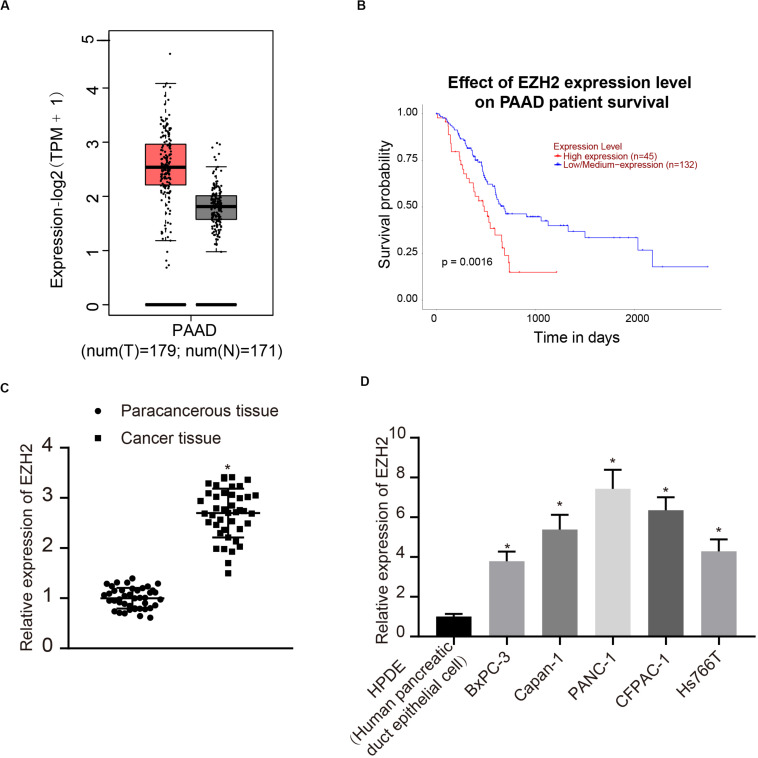
EZH2 overexpression in PC tissues and cells. **(A)** EZH2 expression in PC on the basis of TCGA data. **(B)** EZH2 expression and prognosis of patients with in TCGA PC data. **(C)** Detection of the expression of EZH2 in PC and paracancerous tissues by qRT-PCR, data were expressed by mean ± SD, *n* = 42, paired *t*-test, *compared with paracancerous group, *P* < 0.05. **(D)** Detection of EZH2 expression in different cell lines by qRT-PCR; data were expressed as mean ± SD, *n* = 3, one-way ANOVA and Tukey’s *post hoc* test, *compared with HPDE group, *P* < 0.05.

According to the analysis results of the expression of EZH2 and prognosis of PC in TCGA, EZH2 was highly expressed in tumors, and the survival rate of patients with high expression of EZH2 was significantly lower than that of patients with low expression of EZH2 (Figures 1A,B). Therefore, 42 patients with PC were involved to detect the expression of EZH2 in PC and paracancerous tissues. The results of qRT-PCR showed that the expression of EZH2 in PC was significantly higher than that in paracancerous tissues (*P* < 0.05; Figure 1C). Furthermore, the expression of EZH2 was detected in HPDE cells and several PC cell lines. EZH2 expression in BxPC-3, Capan-1, PANC-1, CFPAC-1, and Hs766T was significantly higher than that in HPDE cells, with the highest expression level in PANC-1. PANC-1 was thus selected for the following experiment (*P* < 0.05; Figure 1D).

### Role of EZH2 on the Proliferation, Migration and Aerobic Glycolysis of PC Cells

**FIGURE 2:**
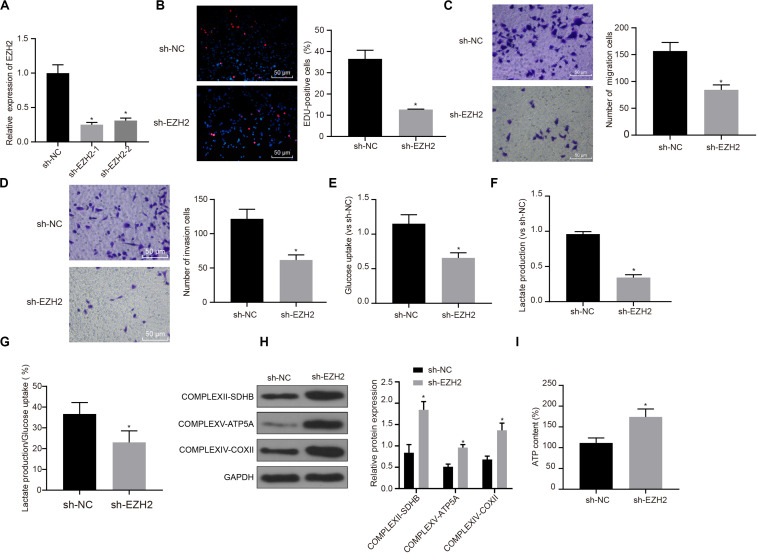
Inhibitory effect of interference with EZH2 on the proliferation, migration and aerobic glycolysis of PC cells, and promoting effect on the recovery of mitochondrial oxidative phosphorylation. **(A)** Detection of transfection efficiency of EZH2 by qRT-PCR. **(B)** Detection of the proliferation of PC cells after interference with EZH2 by EdU. **(C)** Detection of the migration of PC cells after interference with EZH2 by Transwell assay. **(D)** Detection of the invasion of PC cells after interference with EZH2 by Transwell assay. **(E)** Detection of glucose consumption in PC cells after interference with EZH2 by hexokinase method. **(F)** Detection of lactate production in PC cells after interference with EZH2 by hexokinase method. **(G)** Proportion of glycolysis pathways in glucose metabolism. **(H)** Detection of the expression of key proteins of mitochondrial complex in each group by western blot. **(I)** Detection of ATP in supernatant of PC cells with ATP Kit. Data were expressed as mean ± SD, unpaired Student’s *t*-test, *n* = 3, *compared with sh-NC group, *P* < 0.05.

Interference treatment on EZH2 was carried out in PC cells to further explore whether EZH2 can affect the function of PC cells, and sequences with the best interference efficiency was selected for subsequent experiments (*P* < 0.05; Figure 2A). The proliferation, migration and invasion of cells in each group were detected by EdU and Transwell assay. Compared with sh-NC group, the proliferation, migration and invasion ability of sh-EZH2 group decreased significantly (*P* < 0.05; Figures 2B–D), suggesting that EZH2 could inhibit the proliferation, migration and invasion of PC cells.

In order to study the effect of EZH2 on the metabolism of PC cells, EZH2 expression was silenced in PANC-1 cells, and then glycolysis and mitochondrial oxidative phosphorylation were detected in each group of PANC-1 cells. By using hexokinase method, glucose uptake and lactate production of PC cells were significantly reduced after EZH2 silencing (*P* < 0.05; Figures 2E,F), suggesting that EZH2 could inhibit the glycolysis of PC cells. Meanwhile, the proportion of glucose used for glycolysis in the total glucose intake was calculated according to the amount of lactate production. When EZH2 was silenced, the proportion of glycolytic glucose decreased in total glucose intake (*P* < 0.05; Figure 2G).

According to western blot results of key protein expression of mitochondrial complex, the expression of COMPLEXII-SDHB, COMPLEXV-ATP5A and COMPLEXIV-COXII increased significantly in sh-EZH2 group compared with sh-NC group (*P* < 0.05; Figure 2H). The results showed that EZH2 silencing could restore the expression of mitochondrial complex key proteins. At the same time, ATP in PC cells of each group was measured and the level of ATP in PC cells increased significantly after EZH2 silencing (*P* < 0.05; Figure 2I). It revealed that EZH2 can inhibit abnormal aerobic glycolysis of PC cells, restore mitochondrial oxidative phosphate production, and reverse abnormal glucose metabolism of PC cells.

### Recruitment of lncRNA BLACAT1 on EZH2 Regulating the Function of PC Cells

**FIGURE 3:**
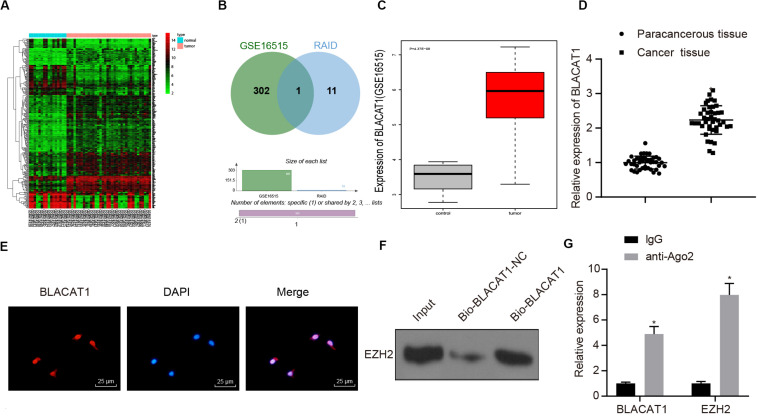
Recruitment effect of lncRNA BLACAT1 on EZH2 to regulate the function of PC cells. **(A)** EZH2 gene expression in PC on the basis of TCGA, the abscissa represents sample type, the ordinate represents expression value, the red box represents normal sample, and the gray box represents tumor sample. **(B)** Intersection of the results of lncRNA prediction in the upstream of EZH2 with the differential expressed gene of GSE16515 chip. The middle part represents the intersection of the two sets of data. **(C)** Differential expression of BLACAT1 in GSE16515 chip, the abscissa represents the sample type, the ordinate represents the expression, the red box represents the tumor sample, the gray box represents the normal sample, and the upper left is the corrected difference *P*-value. **(D)** Detection of BLACAT1 expression in PC tissues and paracancerous tissues by qRT-PCR. **(E)** Subcellular localization of BLACAT1 by FISH assay. **(F)** RNA pull down experiment to observe whether BLACAT1 can bind to EZH2. **(G)** Detection of the binding between BLACAT1 and EZH2 by RIP assay. Data were expressed as mean ± SD, unpaired Student’s *t*-test, *n* = 3, *P < 0.05.

To clarify the upstream regulatory mechanism of EZH2, GSE16515 chip was obtained through GEO database, including 16 normal samples and 36 PC samples. Gene differential expression analysis showed that there were 303 genes with significant differential expression in PC (Figure 3A). Meanwhile, relevant lncRNA in the upstream of EZH2 was predicted by RAID database, and the intersection between the predicted results and chromatin immunoprecipitation (ChIP) analysis results was obtained (Figure 3B). Only one lncRNA was found in the intersection, which was BLACAT1 that was highly expressed in PC (Figure 3C). According to the results of BLACAT1 expression in PC and paracancerous tissues by qRT-PCR, the expression of BLACAT1 in PC tissues was significantly higher than that in paracancerous tissue (*P* < 0.05; Figure 3D). The subcellular localization of BLACAT1 was predicted by searching Bioinformatics website http://lncatlas.crg.eu/. Meanwhile, the subcellular localization of BLACAT1 was detected by FISH experiment. The results showed that BLACAT1 located mainly in the nucleus (Figure 3E). The RNA pull down experiment was used to observe whether BLACAT1 can bind to EZH2. Western blot revealed a successful detection of the presence of EZH2 protein, which indicated that EZH2 could interact with BLACAT1 (Figure 3F). The results of RIP showed that the existence of BLACAT1 could be detected by qRT-PCR after RNA was extracted from the protein solution and pulled down by EZH2 antibody, which further verified the binding between BLACAT1 and EZH2 (Figure 3G).

### BLACAT1 Has Similar Function as EZH2 in PC Cells

**FIGURE 4:**
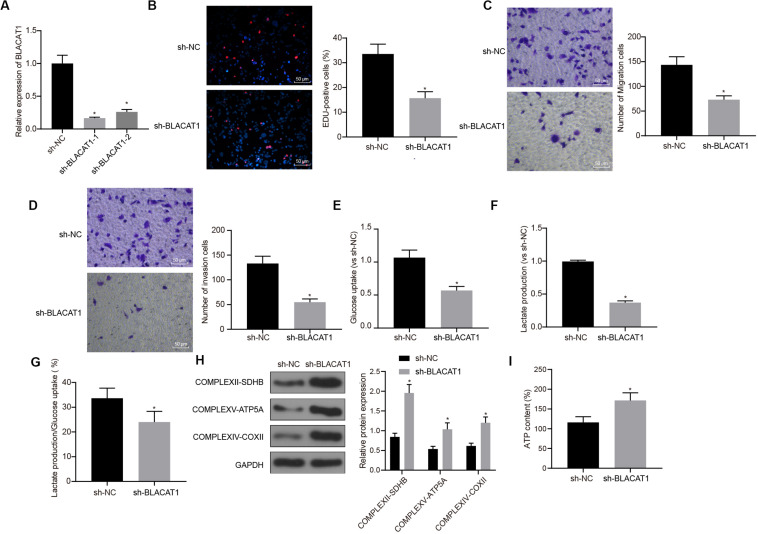
Inhibitory effect of interference with BLACAT1 on the proliferation, migration and aerobic glycolysis of PC cells, and promoting effect on the recovery of mitochondrial oxidative phosphorylation. **(A)** Detection of transfection efficiency of BLACAT1 by qRT-PCR. **(B)** Detection of the proliferation of PC cells after interference with BLACAT1 by EdU assay. **(C)** Detection of the migration of PC cells after interference with BLACAT1 by Transwell assay. **(D)** Detection of the invasion of PC cells after interference with BLACAT1 by Transwell assay. **(E)** Detection of glucose consumption in PC cells after interference with BLACAT1 by hexokinase method. **(F)** Detection of lactate production in PC cells after interference with BLACAT1 by hexokinase method. **(G)** Proportion of glycolysis pathways in glucose metabolism. **(H)** Detection of the expression of key proteins of mitochondrial complex in each group by western blot. **(I)** Detection of ATP in supernatant of PC cells with ATP Kit. Data were expressed as mean ± SD, unpaired Student’s *t*-test, *n* = 3, *compared with sh-NC group, *P* < 0.05.

In order to observe the effect of BLACAT1 on PC cells, after interference of BLACAT1 (Figure 4A), the proliferation, migration and invasion of cells in each group were detected by EdU and Transwell assays. Compared with sh-NC group, the proliferation, migration, and invasion decreased significantly in sh-BLACAT1 group (*P* < 0.05; Figures 4B–D). Meanwhile, the glucose uptake and lactate production of PC cells decreased significantly after BLACAT1 was silenced (*P* < 0.05; Figures 4E,F). Meanwhile, compared with sh-NC group, the proportion of glycolysis glucose in total glucose intake decreased in sh-BLACAT1 group (*P* < 0.05; Figure 4G). According to western blotting results for the detection of the expression of key proteins of mitochondrial complex in each group, the expression of COMPLEXII-SDHB, COMPLEXV-ATP5A, and COMPLEXIV-COXII increased significantly in sh-EZH2 group than in sh-NC group (*P* < 0.05; Figure 4H). Besides, ATP level in PC cells increased obviously after BLACAT1 silencing (*P* < 0.05; Figure 4I). The above results indicated that interference with BLACAT1 expression inhibited the proliferation, migration and aerobic glycolysis of PC cells, and promoted the recovery of mitochondrial oxidative phosphate capacity.

### Recruitment of EZH2 by BLACAT1 to the Promoter Region From EZH2 to Inhibit the Expression of CDKN1C

Furthermore, the downstream mechanism of EZH2 was searched. It was found that EZH2 could promote H3K27 trimethylation of CDKN1C (51, 52).

**FIGURE 5:**
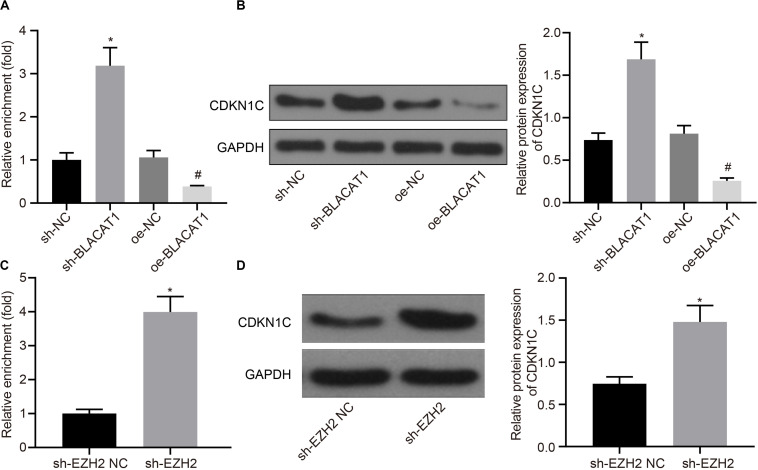
Recruitment of EZH2 by BLACAT1 to the promoter region from EZH2 to inhibit the expression of CDKN1C. **(A)** Detection of H3K27me3 enrichment in CDKN1C promoter by ChIP experiment. **(B)** Detection of CDKN1C expression in PC cells by western blot. **(C)** Detection of H3K27me3 enrichment in CDKN1C promoter by ChIP experiment. **(D)** Detection of CDKN1C expression after EZH2 knockdown in PC cells by western blot. Data were expressed as mean ± SD, unpaired Student’s *t*-test, *n* = 3, *P < 0.05. *Compared with sh-EZH2 NC group, *P* < 0.05; ^#^compared with oe-NC group, *P* < 0.05.

According to the results of ChIP assay after interference with and overexpressing BLACAT1 in PC cells, it was found that enrichment of H3K27me3 in the promoter region of CDKN1C increased significantly in sh-BLACAT1 group than that in sh-NC group. Besides, enrichment of H3K27me3 in the promoter region of CDKN1C was significantly inhibited in oe-BLACAT1 group than that in oe-NC group (*P* < 0.05; Figure 5A). western blot was used to detect CDKN1C expression in PC cells after interference with BLACAT1 and overexpression of BLACAT1. The results showed that the expression of CDKN1C in sh-BLACAT1 group was much higher than that in sh-NC group, and the expression of CDKN1C in oe-BLACAT1 group was evidently lower than that in oe-NC group (*P* < 0.05; Figure 5B). Furthermore, in view of the results of ChIP assay after EZH2 knockdown in PC cells, the enrichment of H3K27me3 in the promoter region of CDKN1C increased significantly in sh-EZH2 group when compared with that in sh-EZH2 NC group (*P* < 0.05; Figure 5C). Besides, western blot was performed to detect CDKN1C expression after EZH2 knockdown in PC cells. Compared with sh-EZH2 NC group, the expression of CDKN1C increased significantly in sh-EZH2 group (*P* < 0.05; Figure 5D). It suggested that BLACAT1 could promote the trimethylation of H3K27 by recruiting EZH2 to the promoter region of CDKN1C, thus inhibiting the expression of CDKN1C.

### Effect of Interference With BLACAT1 to Promote the Expression of CDKN1C on the Proliferation, Migration, and Aerobic Glycolysis of PC Cells, and on the Recovery of Mitochondrial Oxidative Phosphorylation by Stimulating the Expression of CDKN1C

**FIGURE 6:**
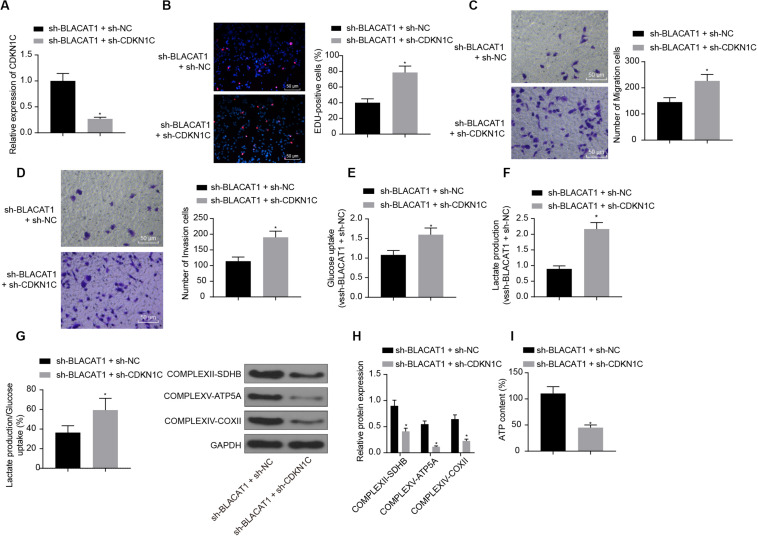
Effect of interference with BLACAT1 to promote the expression of CDKN1C on the proliferation, migration and aerobic glycolysis of PC cells, and on the recovery of mitochondrial oxidative phosphorylation by stimulating the expression of CDKN1C. **(A)** Detection of CDKN1C expression by qRT-PCR. **(B)** Detection of proliferation of PC cells by EdU test. **(C)** Detection of the migration of PC cells by Transwell assay. **(D)** Detection of the invasion of PC cells by Transwell assay. **(E)** Detection of glucose consumption in PC cells by hexokinase method. **(F)** Detection of lactate production in PC cells by hexokinase method. **(G)** Proportion of glycolysis pathways in glucose metabolism. **(H)** Detection of the expression of key proteins of mitochondrial complex in each group by western blot. **(I)** Detection of ATP in supernatant of PC cells with ATP Kit. Data were expressed as mean ± SD, unpaired Student’s *t*-test, *n* = 3, *compared with sh-BLACAT1 + sh-NC group, *P* < 0.05.

In order to detect the influence of BLACAT1 on the development of PC by regulating the expression of CDKN1C, both BLACAT1 and CDKN1C were interfered in PC cells. qRT-PCR was performed to detect the expression of CDKN1C. In comparison with sh-BLACAT1 + sh-NC group, sh-BLACAT1 + sh-CDKN1C group had significant decreased expression of CDKN1C (*P* < 0.05; Figure 6A). Subsequently, EdU test and Transwell assay detected the proliferation, migration and invasion of cells in each group. Compared with sh-BLACAT1 + sh-NC group, the proliferation, migration, and invasion of PC cells increased significantly in sh-BLACAT1 + sh-CDKN1C group (Figures 6B–D). Meanwhile, glucose uptake and lactate production of PC cells were significantly increased (Figures 6E,F), and the proportion of glycolytic glucose in total glucose uptake was increased (*P* < 0.05; Figure 6G). Western blot was then performed to test the expression of key proteins of mitochondrial complex in each group. Compared with control group, sh-BLACAT1 + sh-CDKN1C group had reduced expression of COMPLEXII-SDHB, COMPLEXV-ATP5A and COMPLEXIV-COXII in PC cells (*P* < 0.05; Figure 6H). Besides, ATP level decreased significantly in PC cells (*P* < 0.05; Figure 6I). The results suggested that interference with BLACAT1 to promote the expression of CDKN1C inhibited the proliferation, migration and aerobic glycolysis of PC cells, and promoted the recovery of mitochondrial oxidative phosphate production.

### High Expression of CCNE in PC and Inhibition of CCNE Expression by CDKN1C

**FIGURE 7:**
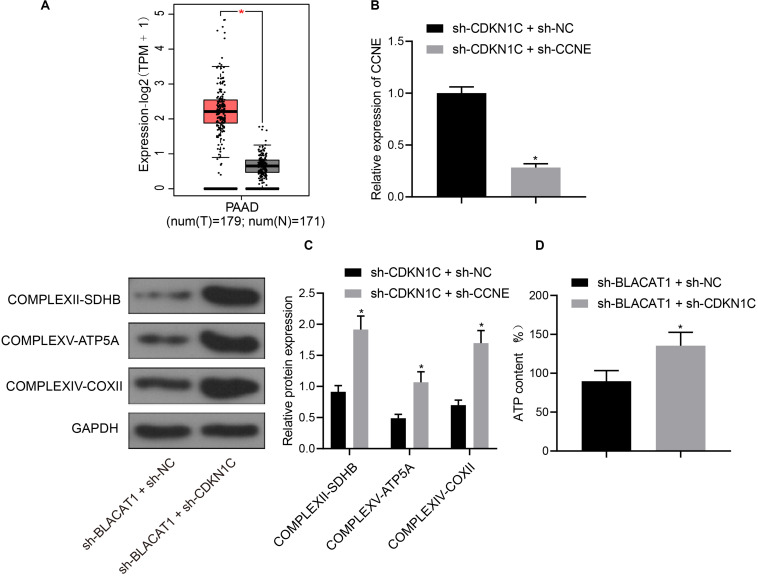
High expression of CCNE in PC and inhibition of CCNE expression by CDKN1C. **(A)** Analysis of CCNE expression in PC through TCGA database. **(B)** Detection of CCNE expression by qRT-PCR. **(C)** Detection of key protein expression of mitochondrial complex in each group by western blot. **(D)** Detection of ATP in supernatant of PC cells with ATP Kit. Data were expressed as mean ± SD, unpaired Student’s *t*-test, *n* = 3, *compared with sh-CDKN1C + sh-NC group, *P* < 0.05.

Furthermore, CDKN1C related pathways and genes were searched in KEGG database, and it was found that CDKN1C could regulate the expression of CCNE (map04110). Further searching of CCNE expression in PC through TCGA database revealed that it was significantly overexpressed in PC (Figure 7A). Simultaneous interference treatment of CDKN1C and CCNE was applied to further investigate whether CDKN1C could regulate the expression of CCNE and thus affect the glycolysis of PC. According to the results, compared with the sh-CDKN1C + sh-NC group, CCNE expression was obviously decreased in sh-CDKN1C + sh-CCNE group (*P* < 0.05; Figure 7B). Western blot was then used to detect the expression of key proteins of mitochondrial complex in each group. Compared with sh-CDKN1C + sh-NC group, there was significant increase in the expression of COMPLEXII-SDHB, COMPLEXV-ATP5A, and COMPLEXIV-COXII in sh-CDKN1C + sh-CCNE group (*P* < 0.05; Figure 7C). Meanwhile, ATP level increased significantly in PC cells (*P* < 0.05; Figure 7D). The above results revealed that CDKN1C could inhibit the expression of CCNE and the glycolysis of PC cells.

### Up-Regulation of CDKN1C Expression to Inhibit Tumor Growth *in vivo* by Interference With BLACAT1

**FIGURE 8:**
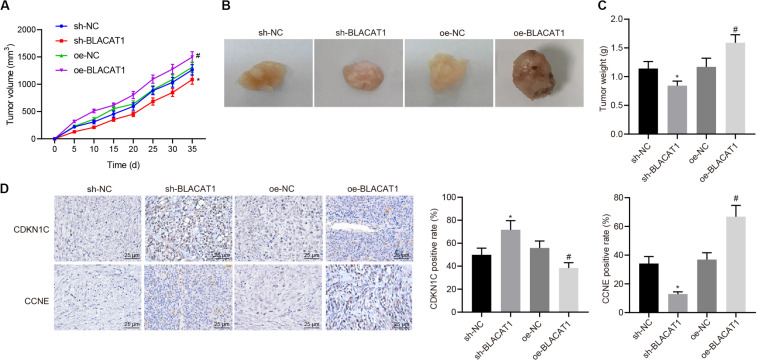
Up-regulation of CDKN1C expression to inhibit tumor growth *in vivo* by interference with BLACAT1. **(A)** Tumor growth curve of each group. **(B)** Visual image of tumor in each group. **(C)** Statistical analysis of tumor mass of each group. **(D)** Immunohistochemical detection of CDKN1C and CCNE expression in tumor tissue. Data were expressed as mean ± SD, unpaired Student’s *t*-test, *n* = 8, *compared with sh-NC group, *P* < 0.05; #compared with oe-NC group, *P* < 0.05.

Subcutaneous tumorigenicity test in nude mice was carried out to detect the tumorigenicity of PC cells. The results showed that compared with sh-NC group, tumor growth rate and tumor mass in sh-BLACAT1 group decreased significantly. Compared with oe-NC group, the growth rate and weight of tumor increased significantly in oe-BLACAT1 group (*P* < 0.05; Figures 8A–C). Besides, based on the immunohistochemical detection results of CDKN1C and CCNE expression in tumor tissue, and it was discovered that in comparison with sh-NC group, the expression of CDKN1C was highly increased and CCNE was decreased in sh-BLACAT1 group. In addition, compared with oe-NC group, oe-BLACAT1 group had evident decreased expression of CDKN1C and increased expression of CCNE (*P* < 0.05; Figure 8D). These results suggested that interference with BLACAT1 can up-regulate CDKN1C expression and inhibit tumor growth *in vivo*.

## Discussion

It has been accepted that lncRNA has multiple functions, which is manifested in the following aspects, such as inducing the transcription by the upstream promoter of protein encoded genes to interfere with the expression of downstream gene; affecting downstream gene expression by inhibiting RNA polymerase II or mediating chromatin remodeling and histone modification; forming complementary double chains with transcripts of protein encoded genes to interfere with mRNA shearing and producing different shearing forms; binding specific proteins and regulating corresponding protein activity as a “molecular bait”; etc. (53–55). In this regard, lncRNA may have major roles in the progression of human tumors via various mechanisms involving the above. It has been reported that there is abnormal expression of lncRNAs in human PC (56–58). However, at present, there are not many conclusive lncRNA in PC, which need further study.

Our study emphasized on the role of lncRNA BLACAT1 regulating EZH2 in PC cells. BLACAT1 is highly expressed in PC by GSE16515 chip data, and prior evidence has pointed out that BLACAT1 can regulate tumor development by binding EZH2 (59). Blocking the expression of BLACAT1 significantly induce the expression of COMPLEXII-SDHB, COMPLEXV-ATP5A, and COMPLEXIV-COXII, correlated with increased ATP level, which indicates the role of BLACAT in regulating PC cell proliferation and aerobic glycolysis by modulating EZH2.

EZH2 can enhance cell proliferation and carcinogenicity, which is highly expressed in human malignancies. EZH2 overexpression was found initially in hematological malignancies (60).

In our study, it was detected that there was EZH2 overexpression in PC tissues and cells. EZH2 is a H3K27 trimethylase and highly expressed in tumors (61, 62). However, the regulatory mechanism of EZH2 is rarely studied in PC. Accordingly, based on data from TCGA, it was found that EZH2 was highly expressed in tumors, and the survival rate of patients with high expression was much lower than that with low expression. qRT-PCR showed that the expression of EZH2 in PC was significantly higher than that in paracancerous tissues, as well as in PC cell lines than that in HPDE. Based on the above, our study validated the inhibitory effect of interference with EZH2 on the proliferation, migration and aerobic glycolysis of PC cells.

The effect of EZH2 in PC was further investigated in our study. We found silencing the expression of EZH2, glucose uptake and lactate production of PC cells were significantly reduced, suggesting that EZH2 could inhibit the glycolysis of tumor cells. The underlying mechanism of EZH2 regulating glycolysis is to restore the expression of mitochondrial complex key proteins. The above results revealed that EZH2 can inhibit abnormal aerobic glycolysis of PC cells, restore mitochondrial oxidative phosphate production, and reverse abnormal glucose metabolism of PC cells.

Meanwhile, our study verified recruitment of EZH2 by BLACAT1 to the promoter region of CDKN1C. It was found that EZH2 could promote H3K27 trimethylation of CDKN1C (51, 52). Silencing both BLACAT1 and CDKN1C silencing resulted in decreased expression of CDKN1C, increased proliferation, migration and invasion, increased glucose uptake, lactate production, and proportion of glycolytic glucose, reduced expression of COMPLEXII-SDHB, COMPLEXV-ATP5A, and COMPLEXIV-COXII, and decreased ATP level. The results suggested that the inhibition of BLACAT1 on CDKN1C expression inhibited the proliferation, migration and aerobic glycolysis of PC cells, and promoted the recovery of mitochondrial oxidative phosphate production.

For further elucidating the mechanism, our study investigated the expression of CCNE in PC and inhibition of CCNE expression by CDKN1C. CCNE has been suggested to be involved in the regulation of glycolysis and other pathways (63, 64). Simultaneous interference with CDKN1C and CCNE decreased CCNE expression, but increased COMPLEXII-SDHB, COMPLEXV-ATP5A, and COMPLEXIV-COXII expression, and increased ATP level. The above results revealed that CDKN1C could inhibit the expression of CCNE and the glycolysis of PC cells. In accordance with the above vivo exploration, we further studied the role of up-regulation of CDKN1C expression to inhibit tumor growth *in vivo* by interference with BLACAT1. The results showed that the growth rate and weight of tumor decreased significantly, the expression of CDKN1C was increased and CCNE was decreased after silencing BLACAT1, but reversed under up-regulating BLACAT1 treatment. It suggested that interference with BLACAT1 can up-regulate CDKN1C expression and inhibit tumor growth *in vivo*.

## Conclusion

**FIGURE 9:**
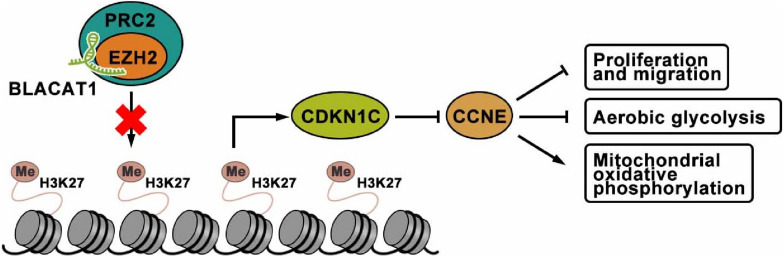
The mechanism of lncRNA-BLACAT1 suppressing the growth of PC cells by inhibiting EZH2 mediated CDKN1C methylation.

To sum up, interference with BLACAT1 in the nucleus can inhibit the H3K27 trimethylation of CDKN1C gene by blocking the recruitment of EZH2 to promote the expression of CDKN1C and inhibit the expression of CCNE, thus suppressing the proliferation, migration and aerobic glycolysis of PC cells, and promoting the recovery of mitochondrial oxidative phosphorylation (Figure 9).

## Data Availability Statement

The original contributions presented in the study are included in the article/supplementary material, further inquiries can be directed to the corresponding author/s.

## Ethics Statement

The studies involving human participants were reviewed and approved by the Ethics Committee of Linyi People’s Hospital. The patients/participants provided their written informed consent to participate in this study. The animal study was reviewed and approved by the Institutional Animal Care and Use Committee of Linyi People’s Hospital.

## Author Contributions

XZ, WG, and HH performed experiments, contributed to experiment design, and assisted in data interpretation. ZJ helped designing experiments, and assisted in data interpretation and statistical analysis. WG and HH supervised planning of experiments and data interpretation, and contributed to the preparation of the manuscript. XZ wrote the manuscript. All authors have read and approved the final submitted manuscript.

## Conflict of Interest

The authors declare that the research was conducted in the absence of any commercial or financial relationships that could be construed as a potential conflict of interest.

## Footnotes

1 http://gepia2.cancer-pku.cn/#index

2 http://ualcan.path.uab.edu/cgi-bin/ualcan-res.pl

3 http://www.rna-society.org/raid2/index.html

4 https://www.ncbi.nlm.nih.gov/geo/

5 https://www.kegg.jp/kegg/pathway.html
